# An in-depth qualitative interview study of female ambulance staff experiences of the menopause transition (CESSATION phase 3)

**DOI:** 10.29045/14784726.2023.12.8.3.20

**Published:** 2023-12-01

**Authors:** Shona Brown, Tessa Noakes, Theresa Foster, Larissa Prothero

**Affiliations:** East of England Ambulance Service NHS Trust ORCID iD: https://orcid.org/0009-0003-6510-2622; East of England Ambulance Service NHS Trust ORCID iD: https://orcid.org/0000-0001-5245-3835; East of England Ambulance Service NHS Trust ORCID iD: https://orcid.org/0000-0002-6395-0885; East of England Ambulance Service NHS Trust ORCID iD: http://orcid.org/0000-0002-5440-8429

**Keywords:** ambulance, female, menopause

## Abstract

**Introduction:**

Menopause is a key workplace issue. Many women will experience symptoms through their later working life. The ambulance service constitutes an employment setting that, dependent on the roles of female staff, can impact on the severity of menopause symptoms and experiences (Prothero et al., 2021). This study aimed to explore female ambulance staff experiences of the menopause transition and suggest ways to improve support offerings.

**Methods:**

A qualitative interpretive approach was adopted, involving 12 UK ambulance services. Participants were identified via purposive sampling, and semi-structured interviews were conducted from February to July 2022 via an online platform or telephone. Recordings were transcribed verbatim and analysed using an inductive thematic approach.

**Results:**

Twenty-two female participants, aged between 42 and 62 years, were interviewed, and represented all phases of the menopause: peri-menopause (n = 9); menopause (n = 5); post-menopause (n = 3); and unsure (n = 5). Fourteen participants had front-line (patient-facing) or emergency operation centre-based roles, while seven were employed in service support roles. Ten themes were identified: impact on work role; awareness and preparedness for menopause transition; personal impact of symptoms; desired support; appropriate sickness and menopause policy; managerial development; compassion and dignity; impact of working environment; impact on safety; and lack of choice. Lack of understanding and support from colleagues and line managers were identified as the key issues. This is included under the managerial development and compassion and dignity themes.

**Conclusions:**

The varying range of menopausal symptoms and their severity impacted on women’s performance at work. The experience of working while going through the menopause could be challenging. Employers should adopt a menopause policy which includes training and awareness for all staff, and suitable for front-line as well as service support staff. There is a need to create a culture where the menopause is not taboo, and women feel able to talk about their symptoms.

## Introduction

The menopause typically occurs between 45 and 55 years of age, and a wide range of menopausal symptoms can be experienced which can have significant personal and work life impacts (Rees et al., 2021). More than one in four women take time off work due to their symptoms. One in 10 women have left their employment, 14% have reduced their working hours and 8% have not pursued promotion (Bazeley et al., 2022).

Appropriate menopause support at work is important not only to promote staff morale, but also to aid the retention of experienced and skilled workers. Lost work productivity related to menopause symptoms results in substantial economic burden (Faubion et al., 2023).

Until recently there has been limited research into women’s health, in particular women’s experiences of menopause at work (Brewis et al., 2017; Department of Health and Social Care, 2022). Previous work has typically focused on menopause symptoms and treatments; however, recent research has included women’s experiences of the menopause in the emergency services, highlighting a need for supportive organisational culture (Atkinson et al., 2021; Prothero et al., 2021).

The CESSATION study was a three-phase mixed-methods exploration of menopause impacts on working life in the ambulance service setting. The aim of this paper is to describe the findings of phase 3 of CESSATION, which comprised semi-structured interviews for an in-depth exploration of female staff experiences of the menopause transition.

## Methods

### Study design

Qualitative research underpinned by phenomenological concepts was considered appropriate to understand the social world from the perspective of the research subject (Denzin & Lincoln, 2005). An inductive approach was adopted to explore experiences and find meaningful insight of female ambulance staff at different phases of the menopause. For CESSATION phase 3, qualitative semi-structured research interviews were undertaken.

### Setting and sampling

Participants completing the national CESSATION phase 2 survey were able to provide an expression of interest to participate in a phase 3 study interview. A total of 400 responses were received from across the 12 participating ambulance services (covering a mixture of urban, rural and coastal areas). Participants were purposively sampled to ensure representation of each menopause transition phase. Participants self-reported whether they were peri-menopausal, menopausal or post-menopausal. Individuals who stated they were ‘unsure’ which phase of the menopause they were experiencing were also sampled. The peri-menopause is defined as the period prior to the cessation of menstruation, and is associated with fluctuating hormones, lasting from a few months to up to 10 years. Menopause is the time when menstruation ceases. Post-menopause is the life phase after which menstruation has ceased for 12 months (National Institute of Clinical Excellence, 2019).

Participant information sheets and consent forms were emailed to participants using their preferred email address provided in their expression of interest. They were given two weeks to respond, with no follow-up contact made. Interviews were arranged at mutually convenient times for participants. On completion of their study interview, participants received a £20 gift voucher as an honorarium for their contribution.

### Data collection

Participants were interviewed by co-author TN or SB between February and July 2022. The semi-structured interviews (see Supplementary 1) were conducted using Microsoft Teams or telephone and recorded digitally using an audio-recording device. Interviews lasted between 20 minutes and one hour. They were transcribed verbatim by co-authors TN and SB using the coding software NVivo, with recordings stored on password-protected East of England Ambulance NHS Trust computers.

### Data analysis

Interviews were analysed by TN and SB using an inductive thematic approach (Braun & Clarke, 2006). The six stages of analysis were followed. The first stage was to familiarise with each transcript content; key points were highlighted. Initial codes were generated independently to reduce bias and add rigour to the findings (Noble & Smith, 2015) before working collaboratively comparing codes. Codes were not pre-conceived and were produced inductively from the data. Themes were found and reviewed after discussion and reflection with the co-authors. The 10 themes previously generated from the CESSATION phase 2 survey were used to promote further discussion. As all authors were female, these discussions facilitated reflection to minimise any pre-existing bias. Transcriptions were not sent to participants for validation due to study budget and time constraints.

### Patient and public involvement

The CESSATION study team included one male and two female patient and public members. Their key involvement was in the development of study materials and interpretation of survey findings.

## Results

**Figure 1:**
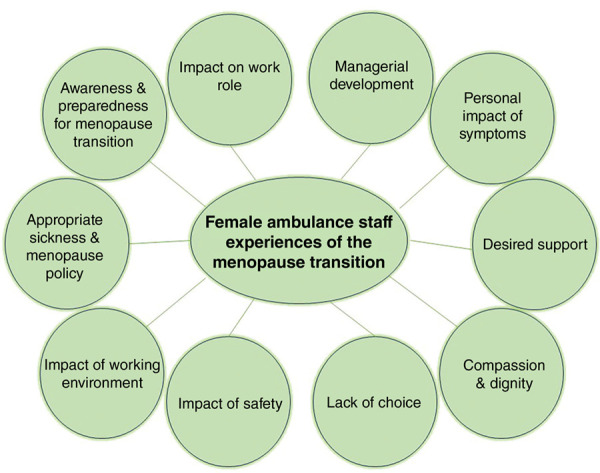
Figure 1. Summary of key interview themes identified in CESSATION phase 3.

Twenty-two semi-structured interviews were completed, with peri-menopause, menopause and post-menopause stages being represented (see Table 1). Ten key themes were identified and are summarised in Figure 1.

**Table 1.** Stage of menopause transition, age and service role of CESSATION interview participants.

Participant ID	Menopause transition phase	Age (years)	Service role	CESSATON ambulance service ID
A	Peri-menopause	50–54	Service support	5
B	Menopause	50–54	Service support	3
C	Peri-menopause	40–44	Operational service delivery – emergency	9
D	Not sure	50–54	Operational service delivery – emergency	12
E	Menopause	50–54	Operational service delivery – emergency	11
F	Not sure	50–54	Would prefer not to state	10
G	Not sure	50–54	Service support	4
H	Post-menopause	50–54	Operational service delivery – emergency	4
I	Not sure	50–54	Operational service delivery – emergency	10
J	Peri-menopause	45–49	Ambulance operations centre	11
K	Peri-menopause	40–44	Ambulance operations centre	1
L	Post-menopause	60–64	Service support	8
M	Peri-menopause	45–49	Service support	3
N	Menopause	50–54	Ambulance operations centre	1
O	Peri-menopause	50–54	Operational service delivery – emergency	6
P	Peri-menopause	50–54	Operational service delivery – emergency	12
Q	Peri-menopause	55–59	Operational service delivery – emergency	2
R	Peri-menopause	50–54	Operational service delivery – emergency	6
S	Post-menopause	50–54	Operational service delivery – emergency	1
T	Menopause	50–54	Operational service delivery – emergency	12
U	Not sure	40–44	Service support	12
V	Menopause	55–59	Service support	12

### Theme 1: impact on work role

Many participants reported their work role had been significantly impacted due to menopause symptoms. Shift patterns could be an issue when managing symptoms, particularly night, 12-hour and full-time shift patterns.

*I couldn’t work with the symptoms I was experiencing because I was all over the place, and I had no control on what was going on.* (Participant D)

Many still expressed concerns about how symptoms affected their ability to perform. Most participants found it challenging to agree changes to their roles with management, and many considered leaving roles due to confidence issues and being unable to manage menopausal symptoms.

*I think sometimes when things get really bad . . . you’re made to kind of change career because a lot of people do leave because they can’t cope anymore.* (Participant M)

### Theme 2: awareness and preparedness for menopause transition

Most participants recognised a lack of awareness and preparedness for the menopause transition, including the range and severity of symptoms experienced.

*I didn’t associate them with menopause at the time . . . I thought there was something else wrong with me.* (Participant U)

Workplace menopause information and awareness for all staff (including men) was highlighted.

*Every woman eventually is going to go through it. And the thing is, every man’s going to go through it . . . if they’re married, they’ve got sisters, they’ve got daughters . . . So, they will, you know, some kind of education around it is better than none.* (Participant A)

This was mirrored in participants’ experiences when approaching management for support, and a lack of awareness among other staff, particularly younger members of the workforce.

### Theme 3: personal impact of symptoms

The personal impact of symptoms varied between participants; however, all noted how the most bothersome symptoms had a considerable effect on both their private and work lives.

*It did disrupt daily life significantly.* (Participant H)

Participants discussed the physical and mental impact of symptoms and how other medical diagnoses were considered prior to menopause. Pre-existing medical conditions were often impacted by the menopause symptoms, adding further concerns for participants.

### Theme 4: desired support

Participants described how they found support sharing personal menopause experiences with women of similar ages in and outside of work. Those who were patient facing recognised the value of empathetic crewmates who offered physical and mental support while on shift.

*Those are the women that I go to for support . . . they just kind of helped me come to terms with that new normal if you like.* (Participant M)

All participants discussed how ambulance services could improve their menopause support, which included support groups, mandatory training and menopause policies. Some participants recognised their service had support opportunities in place, but felt uncomfortable engaging with them, due to embarrassment having to discuss personal issues, male management or service policies not relevant to their role.

### Theme 5: appropriate sickness and menopause policy

Some participants were aware their service had a menopause policy, however those who were front line and patient facing felt it was irrelevant to them and a role-specific policy was warranted. Generic menopause policies were criticised for being only suitable for office staff: the working environment of front-line and patient-facing staff, often considered to be challenging and unpredictable, differs markedly from that of an office environment.

*I think the policy they’ve got, if you’re office based, I’d say it’s fantastic.* (Participant U)

Participants discussed the requirement to take sick leave for menopause symptoms, in the absence of an adequate menopause policy, and highlighted sickness policies did not offer menopausal staff appropriate support. Some noted they had been given no choice but to take long-term sickness leave when experiencing difficult symptoms.

*I just feel that if I’d had other options and with being able to do different duties, short notice change into my hours … I genuinely feel that I wouldn’t have gone off sick.* (Participant Q)

### Theme 6: managerial development

Managers were often seen to lack compassion, empathy and understanding when managing menopausal women. Some participants felt their symptoms were not taken seriously, and accommodations were not made.

*Well, I suggest this isn’t the job for you – go find another job.* (Participant T)

‘Misogynistic’, ‘old boys’ club’, ‘chauvinistic’, ‘male-dominated’ and ‘patriarchal’ were terms used to describe the attitudes and behaviours of some male managers within some services.

Most participants felt they could not approach their manager for support, often because they were male and they felt uncomfortable confiding in them, or concerned their manager may feel awkward and uncomfortable. Discussing embarrassing symptoms, such as flooding, with a male manager was considered too awkward. However, one participant reported her male manager was an older man whose wife had been through the menopause so he was empathetic and supportive. She felt life experience made a difference.

*I’ve had huge support from my manager …. For a male manager to want to take that time and to … show a lot of understanding. It was refreshing.* (Participant M)

There was a general belief that managers needed more menopause awareness training and structured guidance. Often menopause policies were in place, but managers were unaware. One participant described how she had printed her Trust policy ahead of approaching her manager to discuss her symptoms.

### Theme 7: compassion and dignity

Many symptoms experienced by participants caused physical and emotional embarrassment and discomfort. Flooding and hot flushes were two symptoms reported to cause significant problems at work. Lack of understanding meant participants felt they had to ‘just get on with it’ or were unable to confide in anyone.

*When I was having heavy vaginal bleeding, I was left by myself. I had a manager just chuck some wipes under the door at me.* (Participant F)

Humour was often used to deflect or make light of a personal situation. While some participants accepted that banter was part of the service culture and jokes about menopause would be made, others disagreed and felt that experiencing embarrassing symptoms (like hot flushes) were made worse by jokes from colleagues, including managers.

*Hang on to your uterus … we’re going to have to lift [a patient] this first night!* (Participant K)

For women struggling to understand and accept the menopause transition, finding individuals such as colleagues, managers and other healthcare professionals (e.g. GPs) to treat them with dignity and compassion was considered by participants to make a huge difference to their personal well-being.

### Theme 8: impact of working environment

All participants considered control over environment and regular access to toilet facilities the greatest issues when trying to manage menopause symptoms. Office-based participants reported work difficulties despite being able to open windows or use fans when suffering hot flushes. The embarrassment of hot flushes or sweating could impact indoor-working and meeting attendance.

*it’s debilitating and embarrassing.* (Participant K)

Regular access to toilets and changing and showering facilities was identified as a key issue for front-line participants, particularly when experiencing heavy bleeding and intense hot flushes. The wearing of uniform or personal protective equipment and lack of or faulty ambulance temperature control exacerbate flushing symptoms.

*I was absolutely saturated in sweat, and had to go off the road … and have a shower and change my uniform.* (Participant C)

Participants working in offices acknowledged the difficulties faced by front-line colleagues.

*Now working from home, it’s much easier to manage. If I’m hot I’ll open a window and not worry about anybody shouting and sort of, not understanding it.* (Participant B)

### Theme 9: impact on safety

All front-line participants expressed concerns for patient safety while experiencing menopausal symptoms, in particular tiredness and ‘brain fog’.

*There were definitely shifts where I didn’t feel safe because I was so tired and couldn’t think clearly.* (Participant A)

Their 12-hour shifts and late finishes meant these participants struggled to obtain adequate rest. They reported that sleep quality was impacted by insomnia, hot flushes, heavy bleeding and bladder problems. One ambulance operation centre participant worried her tiredness could lead to a mistake when taking an emergency call and impact patient care.

### Theme 10: lack of choice

Accepting personal body changes and transitioning to the next life phase challenged some participants. Being unprepared for the menopause and lacking control over symptoms could cause frustrations and difficulties at work, in particular shift working for front-line participants.

*Certain things were happening to my body that I had no control over.* (Participant C)

All participants shared concerns for the consequences of reduced work performance, with some even feeling if they complained they could easily be replaced at work.

The menopause was recognised by some participants just as part of being a woman, something to get through without the need for preferential treatment. This lack of choice gave some participants determination to deal with their symptoms and to live their lives normally.

*I just plodded on, kept my mouth shut and just got on with it.* (Participant I)

## Discussion

The findings of this study offer an in-depth insight into workplace experiences of women experiencing the menopause transition in UK ambulance services.

Women can have very different experiences of menopause in the workplace, depending on individual characteristics and organisational context (Hardy et al., 2018). Working within the ambulance service has pressures and constraints that may cause additional stress and negative experiences for female staff experiencing the menopause.

Variation in menopause awareness and support in the ambulance setting has been highlighted, and most study participants had negative experiences of the menopause while at work. Many felt under-prepared, and this contributed to how they came to terms with their hormonal-based body changes and menopausal symptoms. Lack of understanding, empathy and support from colleagues and managers exacerbated difficulties experienced by participants.

Despite recent improvements in public menopause awareness-raising in the United Kingdom, the menopause is reported to be a taboo subject in the workplace (Beck et al., 2020; Hardy et al., 2018). The ‘Women’s health strategy for England’ (Department of Health and Social Care, 2022) highlighted the importance of menopause support in the workplace, not only for staff well-being but also for staff productivity and retention. As has been found in this study, the menopause is often described as ‘taboo’, making support of women in the workplace more difficult. This was highlighted by participants needing to utilise sick leave or reducing work hours during their menopause transition.

There does appear to be scope for improved ambulance management menopause awareness and education: participants in this study reported service managers to be lacking menopause knowledge, including the impact of symptoms. Many participants felt undervalued and replaceable because of the symptom-based impact on work performance. Further, in agreement with Watkins et al. (2019), due to lack of awareness and communication, many participants felt unable to approach male colleagues and often sought support from multiple managers before finding one who could help.

### Recommendations and future research

The findings of the CESSATION study in their entirety (phases 1 and 2 are currently being prepared for publication) are to be presented and reviewed by a stakeholder panel, convened to identify and prioritise future workstreams and research activities.

### Limitations

It is acknowledged that: (a) interview participants were identified from the CESSATION phase 2 survey, so individuals who did not participate in this survey were not eligible to participate in an interview; (b) the number of participants representing each menopause phase is small so the transferability of the findings at phase level may be limited; and (c) this study occurred after the COVID-19 pandemic: COVID has been found to impact menopause diagnosis and symptoms (Newson et al., 2021; Stewart et al., 2021).

## Conclusion

Women’s experiences of workplace support within ambulance services across the United Kingdom is variable, and there is a need for improved awareness, understanding and support among all staff. This could be achieved through improved policies, staff and manager menopause training and promotion of work-based menopause-focused discussions.

## Author contributions

All named authors contributed to the data collection, analysis and reporting of findings. EQUATOR Network reporting guidelines and the SRQR checklist provided a framework for the preparation of this manuscript. LP acts as the guarantor for this article.

## Conflict of interest

LP is a member of the editorial board of the *British Paramedic Journal*.

## Ethics

HRA approval was gained (reference: 21/HRA/4564) but Research Ethics Committee was not needed due to the involvement of NHS staff only. All participants provided written informed consent prior to participation.

## Funding

The study received funding from the College of Paramedics Small Grant Award 2020 and University of East Anglia Health and Social Care Partners.
